# The Use of Beta Blockers in Takotsubo Syndrome as Compared to Acute Coronary Syndrome

**DOI:** 10.3389/fphar.2020.00681

**Published:** 2020-05-14

**Authors:** Marvin Kummer, Ibrahim El-Battrawy, Thorsten Gietzen, Uzair Ansari, Michael Behnes, Siegfried Lang, Xiaobo Zhou, Martin Borggrefe, Ibrahim Akin

**Affiliations:** ^1^First Department of Medicine, Medical Faculty Mannheim, University Heidelberg, Mannheim, Germany; ^2^DZHK (German Center for Cardiovascular Research), Partner Site, Heidelberg-Mannheim, Mannheim, Germany

**Keywords:** Takotsubo syndrome (TTS), acute coronary syndrome, beta-blockers, long-term mortality, ace-inhibitors

## Abstract

**Background:**

Takotsubo syndrome (TTS) and acute coronary syndrome (ACS) patients have a similar mortality rate. In this study, we sought to determine the short- and long-term outcome of TTS patients as compared to ACS patients both treated with beta-blockers.

**Objectives:**

In the present study we described the data of 5 years of follow up of 103 TTS and 422 ACS patients both treated with beta-blockers.

**Methods:**

Data from TTS patients were included retrospectively and prospectively, ACS patients were included retrospectively. All retrospectively included patients have been followed up for 5 years. The end point in this study was the occurrence of death.

**Results:**

TTS affected significantly more women (87.4%) than ACS (34.6%) (p < 0.01). TTS patients suffered significantly more often from thromboembolic events (14.6% versus 2.1%; p < 0.01) and cardiogenic shock (11.9% versus 3.6%; p < 0.01) than the ACS group. TTS patients had a significantly higher long-term mortality (within 5 years) as compared to ACS patients (17.5% versus 3.6%) (p < 0.01). Patients of the TTS group compared to the ACS group did not benefit from combination of beta-blockers and ACE-inhibitors in terms of long-term mortality (p < 0.01). As we compare TTS patients who were treated with beta-blockers and ACE-inhibitors versus single use of beta-blockers there was no difference in long-term mortality (p = 0.918).

**Conclusion:**

TTS patients had a significantly higher long-term mortality (within 5 years) than patients with an ACS.

## Introduction

It has been reported that Takotsubo (TTS) patients have a similar mortality rate to acute coronary syndrome (ACS) (Redfors et al., 2015). In the acute phase, the clinical presentation, electrocardiographic findings and biomarker profiles are often similar to those of an ACS (Templin et al., 2015). There are different complications which have been reported in connection to TTS, such as cardiogenic shock, sudden cardiac arrest, thromboembolic events, mitral valve regurgitation, and atrial fibrillation (Stiermaier et al., 2015; El-Battrawy et al., 2016; El-Battrawy et al., 2017a; El-Battrawy et al., 2017b; El-Battrawy et al., 2018a), and also have been reported in connection to ACS (Lavie and Gersh, 1990; Baja et al., 2015; Behnes et al., 2018).

Studies have revealed that there is no significant difference in the first 30 d and 1-year mortality between TTS patients who were mostly treated with beta-blocker (carvedilol) and those who were not (Templin et al., 2015; Isogai et al., 2016). On the other hand Yasar et al. conclude in their meta-analysis that beta-blocker therapy is indicated in most of the TTS patients (Sattar et al., 2020).

In contrast, it is well proven that ACS patients benefit of beta-blocker treatment (Ozasa and Kimura, 2019). The current European Society of Cardiology (ESC) guideline for ACS without persisting ST-elevation gave beta-blockers as a class I recommendation (Roffi et al., 2016).

In the present study, we sought to determine the short- and long-term outcome of TTS patients as compared to ACS patients both treated with beta-blockers.

## Methods

### Study Design and Data Source

In this observational cohort study 133 consecutive patients presenting with TTS in the Clinic for Cardiology in the University Hospital Mannheim from 2003 to 2016 were included and followed up retrospectively and from 2017 ongoing prospectively in the study under consistent follow up of complications and mortality. Five hundred twenty-two patients with ST-elevation myocardial infarction (STEMI) and/or non-ST-elevation myocardial infarction (NSTEMI) in the same hospital from 2007 to 2008 were included and followed up retrospectively.

### Study Cohort

All retrospectively included patients have been followed up for 5 years.

The groups were screened for beta-blocker treatment on discharge, so 103 patients with TTS and 422 patients with ACS were included in the calculations.

TTS was defined based on the Mayo clinic criteria (Stiermaier et al., 2015). To validate the diagnosis of TTS, the angiograms, the echocardiograms, and ECGs were reviewed by two independent experienced cardiologists. ACS was defined after the guidelines of the European Society of Cardiology (ESC) (Hamm et al., 2011).

### Study Outcomes

Baseline characteristics of demographics, clinical data, laboratory parameters, and in-hospital events (arrhythmias, cardiac rupture, thromboembolic events, pulmonary congestion with use of non-invasive positive-pressure ventilation, intubation, use of a temporary pacemaker, use of inotropic agents, death) were assessed by chart review.

This study was conducted in compliance with the Declaration of Helsinki. The study protocol was approved by the ethics committee of University Medical Centre Mannheim.

### Study End Point

The end point in the study was the occurrence of death in TTS and ACS patients. Short-term mortality was defined as death in the first 30 d after the index event, long-term mortality as death within 5 years of follow up.

### Statistics

Data are shown as means ± standard deviation for continuous variables with a normal distribution, median (interquartile range) for continuous variables with a non-normal distribution, and as frequency (%) for categorical variables. The Kolmogorov–Smirnov test was used to assess normal distribution. Normally or non-normally distributed continuous variables were compared with Student’s t-test and Mann–Whitney U-test, respectively. Categorical variables were compared by chi-squared-test or Fisher’s exact test. Two-tailed Fisher`s exact test was applied in tests with sample size of n=5 or below. Fisher’s exact ratio test was used for calculation of the relative risk for the occurrence of events. Results are shown with 95% confidence intervals. Kaplan-Meier procedure was performed to evaluate group differences by log-rank test. Statistical analysis was performed with SPSS 23.0, a p < 0.05 (two-tailed) was considered statistically significant.

### Limitations

A potential limitation of this study are the data being based on patients from one single center. A second limitation is the lack of control of the compliance of the patients for taking in the prescribed beta-blockers.

## Results

### Baseline Characteristics

TTS and ACS patients had a similar age (67 ± 11 and 66 ± 13 years; p=0.45), however TTS affected significantly more women (87.4%) than ACS (34.6%) (p < 0.01).

TTS patients showed a significantly longer QTc time (481 ms; IQR 130–700) than the ACS group (443 ms; IQR 371–569) (p < 0.01). TTS patients had significantly lower creatinin kinase MB (CKMB) (29 U/L) (p < 0.01) and hemoglobin (12.3 g/dl) (p < 0.01) as compared to the ACS patients (81 U/L; 13.3 g/dl). Initially the TTS group had a significantly lower left ventricular ejection fraction (LVEF) (39%) than the ACS group (51%), but gained till follow-up up to 53%, whereas the ACS patients stayed on the same level.

The medical history of both groups did not show a difference in the occurrence of diabetes mellitus, but there were significantly more patients with chronic obstructive pulmonary disease (COPD) in the TTS group (17.5% versus 6.6%) (p < 0.01) (**Table 1**). A total of 74.9% of the ACS group and 65% of the TTS group were treated with angiotensin-converting-enzyme inhibitors on discharge (p = 0.04). ACS patients were more often treated with platelet inhibitors as ASS (98.1% versus 45.6%) (p < 0.01) or clopidogrel (88.2% vs 6.8%) (p < 0.01) as well as dual platelet inhibition (ASS + clopidogrel) (87.4% versus 6.8%) (p < 0.01). Therefore TTS patients were more often treated with anticoagulation as cumarine (9.7% versus 2.4%) (p < 0.01), heparin (15.5% versus 5.9%) (p = 0.01), or factor Xa inhibitors (4.9% versus 0%) (p < 0.01).

Table 1Baseline characteristics of 103 patients with Takotsubo syndrome (TTS) and 422 patients with acute coronary syndrome (ACS) both treated with ß-antagonists.

**Table:** 

Variables	TTS (n=103)	ACS (n=422)	p value*
**Demographics**			
Age. mean±SD	67±11	66±13	0.45
Female (%)	90 (87.4)	146 (34.6)	**<0.01**
**Symptoms. n (%)**			
Dyspnea	41 (39.8)	100 (23.7)	**<0.01**
Chest pain	57 (55.9)	342 (81.0)	**<0.01**
**Clinicparameter**			
Systolic BP. mmHg	138 (70-220)	141 (0-280)	0.94
Diastolic BP. mmHg	80 (50-151)	80 (0-150)	0.91
Heart rate. bpm	97±23	92±26	0.84
**ECG data. n (%)**			
ST-segment elevation	32 (31.4)	180 (42.8)	**0.04**
Inversed T-waves	90 (91.8)	196 (46.6)	**<0.01**
PQ-interval	161±28	168±37	0.67
QTc (ms)	481 (130-700)	443 (364-688)	**<0.01**
**Laboratory values. mean±SD**			
Troponin I (U/L) (IQR)	81.51 (0.01-2738)	19.36 (0.02-335.2)	0.18
Creatine phosphate kinase (U/L)	344 (39-4478)	1152 (35-20149)	0.78
CKMB (U/L) (IQR)	29 (1-167)	81 (0-970)	**<0.01**
C-Reactive protein (mg/l) (IQR)	41.7 (0.4-386.8)	35.1 (0.0-594.0)	0.31
Hemoglobin (g/dl) (IQR)	12.3 ± 1.9	13.3 ±2.0	**<0.01**
Creatinine (mg/dl) (IQR)	1.07 (0.40-5.56)	1.15 (0.22-12.16)	0.36
**Echocardiography data n (%)**			
LV EF%	39±10	51±12	**<0.01**
LV EF% follow-up	53±11	51±12	0.09
Mitral regurgitation	50 (48.5)	125 (29.6)	**<0.01**
Tricuspid regurgitation	38 (36.9)	58 (13.7)	**<0.01**
**Medical history. n (%)**			
Smoking	31 (30.1)	170 (40.3)	0.06
Diabetes mellitus	25 (24.3)	134 (31.8)	0.14
BMI>25 kg/m²	33 (36.3)	231 (54.7)	**<0.01**
Hypertension	63 (61.2)	290 (68.7)	0.14
COPD	18 (17.5)	28 (6.6)	**<0.01**
Atrialfibrillation	18 (17.5)	54 (12.8)	0.22
History of malignancy	10 (9.7)	26 (6.2)	0.20
**Variables**	**TTS** **(n=103)**	**ACS** **(n=422)**	p value*
**Drugs on admission. n (%)**			
Beta-blocker	41 (41.8)	133 (31.8)	0.06
ACE inhibitor	42 (42.9)	106 (25.2)	**<0.01**
Aldosterone inhibitor	1 (1.0)	2 (0.5)	0.52
Aspirin	31 (31.6)	117 (27.9)	0.56
Therapeutic anticoagulation	10 (10.3)	24 (5.7)	0.10
**Drugs on discharge. n (%)**			
Beta-blocker	103 (100.0)	422 (100.0)	
ACE inhibitor	67 (65.0)	316 (74.9)	**0.04**
Aldosterone inhibitor	2 (1.9)	5 (1.2)	0.55
Aspirin	47 (45.6)	414 (98.1)	**<0.01**
Clopidogrel	7 (6.8)	372 (88.2)	**<0.01**
Aspirin + clopidogrel	7 (6.8)	369 (87.4)	**<0.01**
Cumarine	10 (9.7)	10 (2.4)	**<0.01**
Heparin	16 (15.5)	25 (5.9)	**0.01**
Factor Xa inhibitors	5 (4.9)	0 (0)	**<0.01**

**Table:** 

**Variables**	**TTS** **(n=103)**	**ACS** **(n=422)**	p value*
**Drugs on admission. n (%)**			
Beta-blocker	41 (41.8)	133 (31.8)	0.06
ACE inhibitor	42 (42.9)	106 (25.2)	**<0.01**
Aldosterone inhibitor	1 (1.0)	2 (0.5)	0.52
Aspirin	31 (31.6)	117 (27.9)	0.56
Therapeutic anticoagulation	10 (10.3)	24 (5.7)	0.10
**Drugs on discharge. n (%)**			
Beta-blocker	103 (100.0)	422 (100.0)	
ACE inhibitor	67 (65.0)	316 (74.9)	**0.04**
Aldosterone inhibitor	2 (1.9)	5 (1.2)	0.55
Aspirin	47 (45.6)	414 (98.1)	**<0.01**
Clopidogrel	7 (6.8)	372 (88.2)	**<0.01**
Aspirin + clopidogrel	7 (6.8)	369 (87.4)	**<0.01**
Cumarine	10 (9.7)	10 (2.4)	**<0.01**
Heparin	16 (15.5)	25 (5.9)	**0.01**
Factor Xa inhibitors	5 (4.9)	0 (0)	**<0.01**

*p values for the comparison between TTS and ACS.

ACE, angiotensin-converting-enzyme; ACS, acute coronary syndrome; BMI, body-mass-index; BP, blood pressure; COPD, chronic obstructive pulmonary disease; ECG, electrocardiogram; EF, ejection fraction; IQR, inter quartile range; LVEF, left ventricular ejection fraction; SD, standard deviation; TTS, Takotsubo syndrome.

### In-Hospital Events

TTS and ACS patients stayed on average 5 (0–52) and 3 (0–20) d on the intermediate care unit (ICU). TTS patients got significantly more noninvasive positive-pressure ventilation or intubation (51.5%) and inotropic agents (10.7%) than ACS patients (3.8% versus 3.3%) (p < 0.01). However significantly more patients of the ACS group got a device-implantation (9.2%) as compared to TTS (2.9%) (p = 0.03). TTS patients suffered significantly more from thromboembolic events (14.6% versus 2.1%; p < 0.01) and cardiogenic shock (11.9% versus 3.6%; p < 0.01) than the ACS group (**Table 2**).

**Table 2:** In-hospital events and treatment strategy in Takotsubo syndrome (TTS) and acute coronary syndrome (ACS) patients both treated with ß-antagonists.

Variables	TTS (n=103)	ACS (n=422)	p value*
Life-threatening arrhythmia	8 (7.8)	29 (6.9)	0.74
NPPV and or intubation	53 (51.5)	16 (3.8)	**<0.01**
Inotropic agents	11 (10.7)	14 (3.3)	**<0.01**
Resuscitation	4 (3.9)	15 (3.6)	0.87
Device-Implantation	3 (2.9)	39 (9.2)	**0.03**
Admission to ICU. length of stay (IQR)	5 (0-52)	3 (0-20)	**<0.01**
In-hospital death	0 (0.0)	1 (0.2)	0.62
Thromboembolic events	15 (14.6)	9 (2.1)	**<0.01**
Cardiogenic Shock	12 (11.9)	15 (3.6)	**<0.01**

*p values for the comparison between TTS and ACS; NPPV. Noninvasive positive pressure ventilation; ICU, Intermediate care unit.

### Outcome and Follow-Up

**Figure 1:**
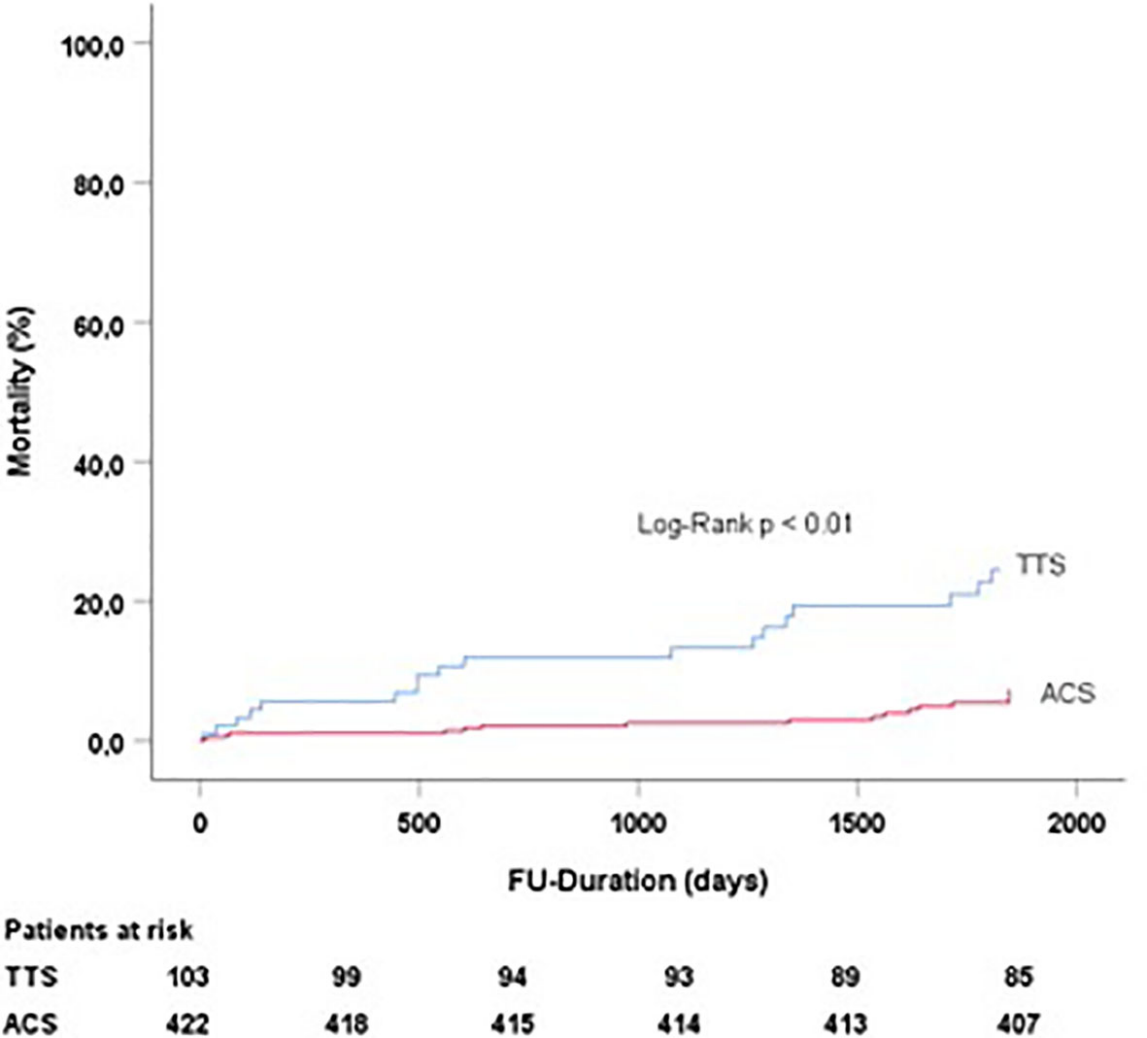
Follow-up duration and mortality in TTS and ACS patients treated with beta blockers. TTS, Takotsubo syndrome; ACS, acute coronary syndrome.

TTS patients had a significantly higher long-term mortality (within 5 years of follow up) as compared to ACS patients (17.5% versus 3.6%) (p < 0.01) (**Figure 1**).

**Figure 2:**
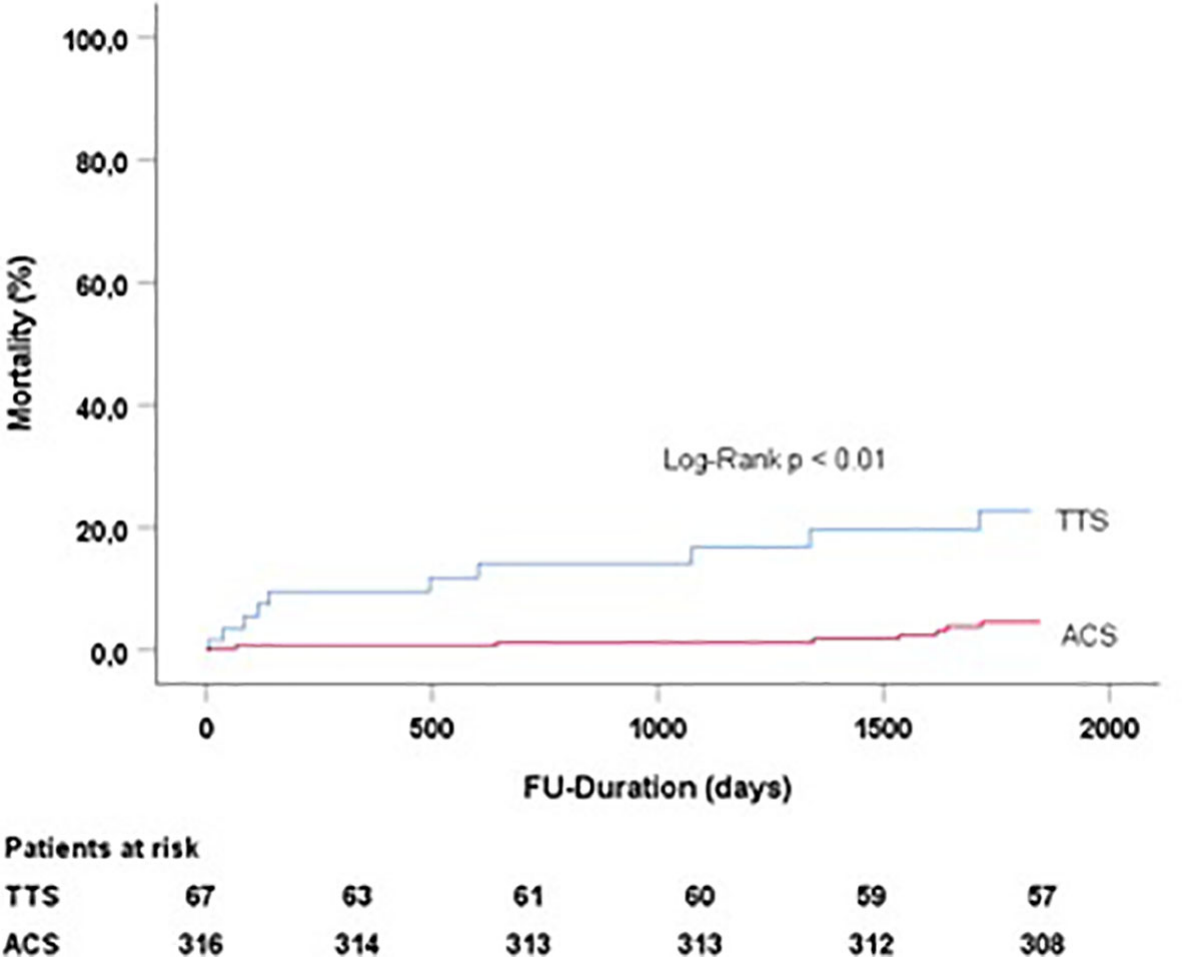
Follow-up duration and mortality in TTS and ACS patients treated with beta-blockers and angiotensin-converting-enzyme inhibitors. TTS, Takotsubo syndrome; ACS, acute coronary syndrome.

Patients of the TTS group compared to the ACS group did not benefit from combination of beta-blockers and angiotensin-converting-enzyme inhibitors in terms of long-term mortality (p < 0.01) (**Figure 2**).

**Figure 3:**
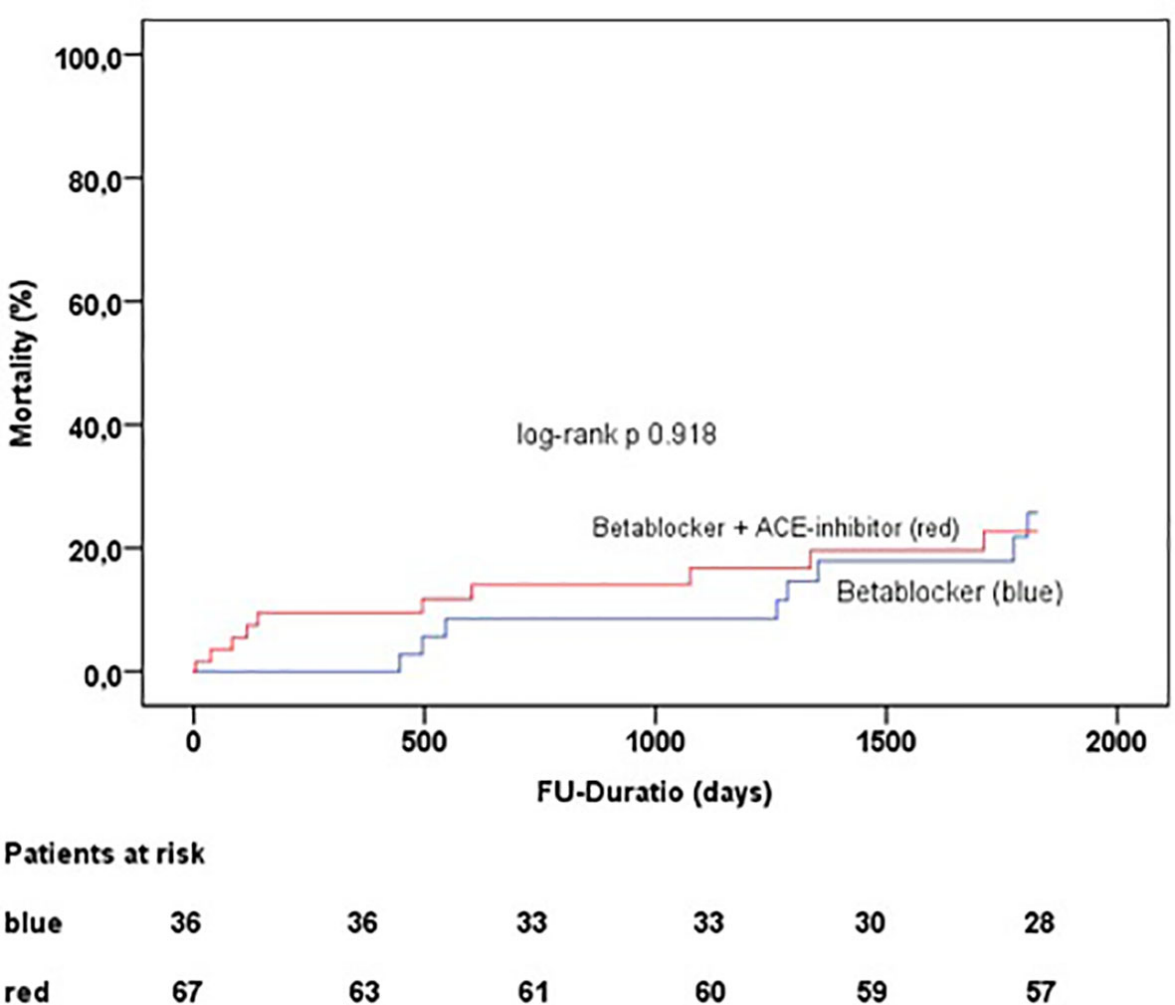
Follow-up duration and mortality in TTS patients treated with beta-blockers and angiotensin-converting-enzyme inhibitors and beta blocker single treatment.

As we compare TTS patients who were treated with beta-blockers and angiotensin-converting-enzyme inhibitors versus single use of beta-blockers there was no difference in long-term mortality (p = 0.918) (**Figure 3**).

ACS patients show significantly more heart failure in long-term follow up compared to the TTS patients (13% versus 2.9%) (p < 0.01) (**Table 3**).

**Table 3:** Outcome in 103 Takotsubo syndrome (TTS) patients and 422 acute coronary syndrome (ACS) patients both treated with ß-antagonists.

Variables	*TTS* (n=103)	*ACS* (n=422)	Relative risk (95% CI)	p value*
In-hospital mortality	0 (0.0)	1 (0.2)		0.62
30-day mortality	1 (1.0)	2 (0.5)	2.0 (0.2-22.4)	0.55
Long-term mortality	18 (17.5)	15 (3.6)	4.9 (2.6-9.4)	**<0.01**
Cardiovascular cause of death	5 (4.9)	14 (3.3)	1.5 (0.5-4.0)	0.45
Non-cardiovascular cause of death	6 (5.8)	1 (0.2)	8.2 (2.1-32.2)	**<0.01**
Unknown cause of death	7 (6.8)	0 (0.0)		**<0.01**
30-day Stroke	4 (3.9)	4 (0.9)	4.1 (1.0-16.1)	**0.03**
Long-term Stroke	6 (5.8)	16 (3.8)	1.5 (0.6-3.8)	0.36
30-day life-threatening arrythmia	7 (6.8)	28 (6.6)	1.2 (0.6-2.5)	0.68
Long-term life-threatening arrythmia	8 (7.8)	32 (7.6)	1.2 (0.6-2.3)	0.69
30-day Heart Failure	2 (1.9)	20 (4.7)	0.4 (0.1-1.7)	0.20
Long-term Heart Failure	3 (2.9)	55 (13.0)	0.2 (0.1-0.7)	**<0.01**
30-day Recurrence	0 (0.0)	6 (1.4)		0.22
Long-term Recurrence	6 (5.8)	46 (10.9)	0.5 (0.2-1.2)	0.12
30-day Thromboembolic Events	15 (14.6)	11 (2.6)	5.6 (2.6-11.8)	**<0.01**
Long-term Thromboembolic Events	19 (18.4)	30 (7.1)	2.6 (1.5-4.4)	**<0.01**

*p values for the comparison between TTS and ACS patients.

### Predictors for Long Term Mortality

Using multivariate Cox regression analysis we determined that LVEF ≤ 35% is a significant predictor for long term mortality (p< 0.01; hazard ratio 3.93; 95% CI 1.83–8.46) even after adjusting for TTS (p = 0.009; hazard ratio 2.75; 95% CI 1.29–5.85), age >75 (p< 0.01; hazard ratio 4.11; 95% CI 1.97–8.59) and cardiogenic shock (p< 0.01; hazard ratio 5.63; 95% CI 2.43–13.06).

## Discussion

We have described the long-term outcome in TTS patients in comparison to ACS patients treated with a concomitant beta-blocker. The main findings in this study

- The TTS group has a higher long-term mortality (within 5 years) than the ACS group and does not seem to profit of the treatment with beta-blockers
- In contrast to earlier studies TTS patients do not seem to profit of the concomitant use of beta-blocker and angiotensin-converting-enzyme inhibitors

As recently published data from the InterTAK registry revealed that TTS patients and patients with ACS have a similar short- and long-term outcome (Templin et al., 2015) we decided to evaluate the long-term outcome of both groups in comparison and taking a closer look on the outcome with both groups with beta-blocker medication on discharge.

TTS mostly occurs in postmenopausal women and is usually provoked by physical or emotional stress (Templin et al., 2015; El-Battrawy et al., 2017c). As one of the main hypotheses concerning the pathophysiology of TTS is that patients suffer from catecholamine-induced cardiotoxicity (Komamura et al., 2014). It may seem to be a good conclusion to treat them with beta-blockers, but we did not find any long-term benefits of this treatment compared to ACS patients with beta-blocker treatment as well.

Using cardiomyocytes from induced pluripotent stem cells (hiPSC-CMs) it has been shown that TTS patients may benefit form beta-blocker treatment as Bochert et al. have shown (Borchert et al., 2017). El-Battrawy et al. have also confirmed a reverse of repolarization changes and shortening of the action potential duration in a TTS model (El-Battrawy et al., 2018b).

Templin et al. showed in their study that the use of angiotensin-converting-enzyme inhibitors medication on discharge was associated with improved survival at 1 year of follow up (Templin et al., 2015). Citro et al. also showed in their recent study that TTS patients with angiotensin-converting-enzyme inhibitors at discharge had lower rates of cardiac death in long-term follow up (Citro et al., 2019). In contrast our study did not show improved long-term survival neither in TTS patients as compared to ACS patients both treated with beta-blockers and angiotensin-converting-enzyme inhibitors, nor in the TTS group itself as comparing combined angiotensin-converting-enzyme inhibitors and beta-blocker treatment versus beta-blocker single use.

Besides the missing effects on long-term mortality in our study Santoro et al. stated in their meta-analysis that neither beta-blockers nor angiotensin-converting-enzyme inhibitors were able to reduce recurrence of TTS (Santoro et al., 2014). As TTS is considered to be often self-limiting and unnecessary treatment should be avoided (Jha et al., 2019) it can be considered to take distance of this therapy regime by the treating physicians.

Citro et al. also showed in their study that TTS patients with LVEF ≤ 35% experienced more in-hospital complications, higher overall mortality and that LVEF ≤ 35% is a predictor for major adverse cardiac events in long-term follow up (Citro et al., 2019). We can confirm these results: By multivariate Cox regression analysis we determined that LVEF ≤ 35% was a predictor for long term mortality (within 5 years) in our cohort even after adjusting for TTS, age >75, and cardiogenic shock.

We have also seen that the TTS patients gained a recovery of LVEF from the initial TTS event till the follow up (**Table 1**), this might be an explanation to the significantly less heart failures in TTS than in ACS patients.

Kim et al. showed in their study that cancer is the most important prognostic factor for death in their followed-up TTS cohort. (Kim et al., 2018) We cannot corroborate these findings. There is no significant difference in history of malignancy in our two cohorts and it does not become statistically significant in univariate Cox regression analysis (p = 0.905; CI 0.251–4.763; hazard ratio (HR) 1.094) in the TTS group.

One reason that beta-blockers are not that efficient in TTS patients as in ACS patients could be that the cardioprotective effect of beta-blockers (Egan et al., 2005) is not that important for TTS patients, because there are significantly more TTS patients dying of non-cardiac causes than ACS patients. Another data point could be the complex pathomechanism of TTS. Patients and physicians also may underestimate their disease and stop the intake of beta-blockers very early after TTS event.

## Conclusion

The long-term mortality in TTS patients is significantly higher within a follow up of 5 years than in ACS patients, both treated with beta-blockers. So we were able to corroborate the findings of earlier studies that TTS patients do not profit of beta-blocker treatment in comparison to ACS patients.

## Limitations

Of note, from the beginning of the patient-recruitment (2003) to the end (2017) the diagnostic standards for TTS in the University Hospital Mannheim have been modified consistent with current guidelines (Lyon et al., 2016). Regarding the concern about the declined outcome of patients, physicians may have been more aware of the diagnosis of TTS. Moreover imaging diagnostic like Cardio-MRI has now been used more often to exclude other possible diagnoses.

## Data Availability Statement

The datasets generated for this study are available on request to the corresponding author.

## Ethics Statement

This study was conducted in compliance with the Declaration of Helsinki. The study protocol was approved by the Ethics Committee of University Medical Centre Mannheim.

## Author Contributions

All authors listed have made substantial, direct, and intellectual contribution to the work and approved it for publication.

## Conflict of Interest

The authors declare that the research was conducted in the absence of any commercial or financial relationships that could be construed as a potential conflict of interest.
